# Supplementary figures and images for: Significance of urine complement proteins in monitoring lupus activity
Source: PeerJ. 2022 Nov 18;10:e14383. doi: 10.7717/peerj.14383 (PMC9677877; doi:10.7717/peerj.14383)

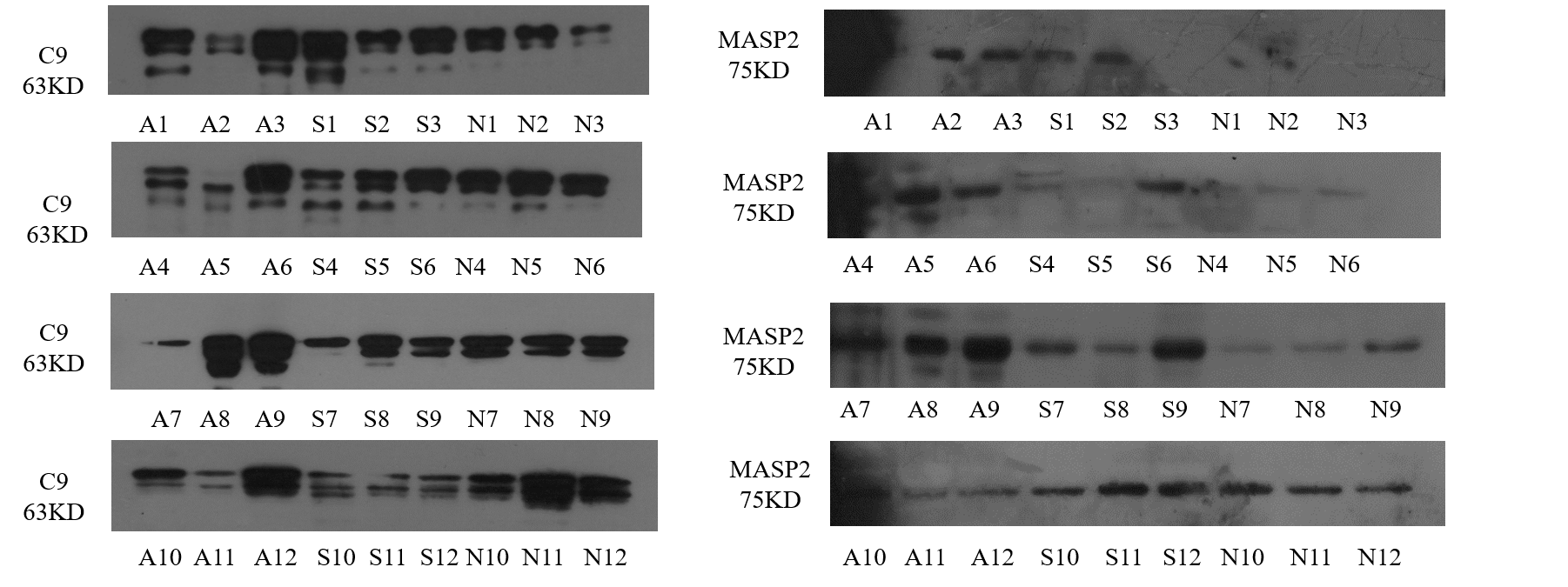

Supplement: Supplemental Information 1 — The total protein were enriched from the urine of each subject. A1-A12: Western blot bands in the urine from 12 SLE-A patients. S1-S12: Western blot bands in the urine from 12 SLE-S patients. N1-N12: Western blot bands in the urine from 12 normal controls. [file peerj-10-14383-s001.png]
